# Supplementary figures and images for: Effect of Yttrium-90 transarterial radioembolization in patients with non-surgical hepatocellular carcinoma: A systematic review and meta-analysis
Source: PLoS One. 2021 Mar 4;16(3):e0247958. doi: 10.1371/journal.pone.0247958 (PMC7932100; doi:10.1371/journal.pone.0247958)

**S1 Figure:** Forest plot of the cumulative risk ratio of dichotomous outcomes.

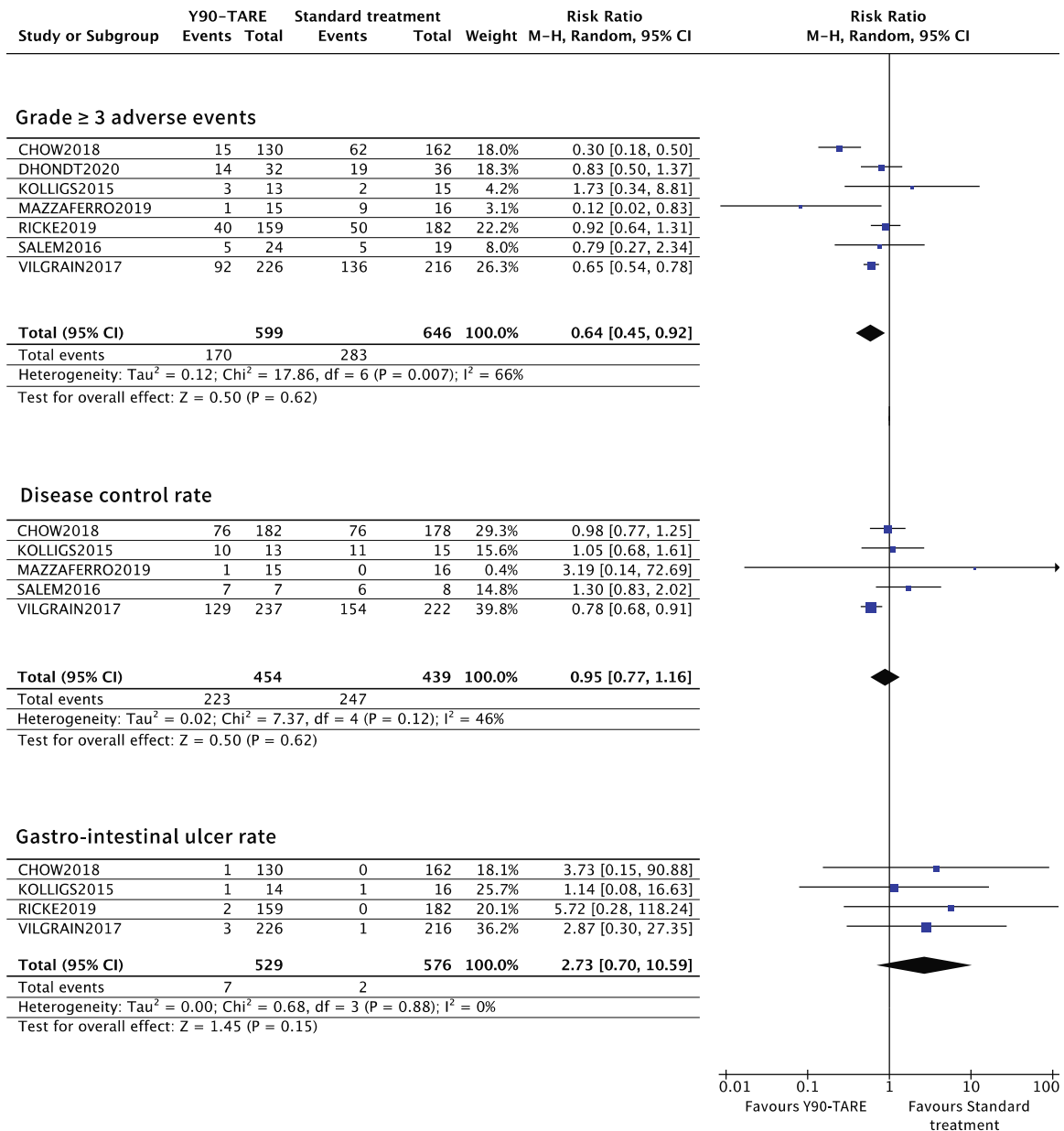

Supplement: S1 Fig — (PDF) [file pone.0247958.s002.pdf]
