# Supplementary material for: Effect of Yttrium-90 transarterial radioembolization in patients with non-surgical hepatocellular carcinoma: A systematic review and meta-analysis
Source: PLoS One. 2021 Mar 4;16(3):e0247958. doi: 10.1371/journal.pone.0247958 (PMC7932100; doi:10.1371/journal.pone.0247958)
Supplement: S1 Table — (DOCX) [file pone.0247958.s003.docx]

**S1 Table:** Summary of findings.

| **Y90-TARE versus standard treatment in patients with non-surgical hepatocellular carcinoma (HCC)**  Population: Patients with non-surgical HCC  Setting: oncologic treatment centers in North America, Pacific-Asia, Turkey and Europe  Intervention: Y90-TARE treatment strategies  Comparison: Standard treatment (TACE and sorafenib) | | | | | | |
| --- | --- | --- | --- | --- | --- | --- |
| Outcomes | Illustrative comparative risk | | Effect estimate  (95% CI) | Number of participants | Quality of the evidence (GRADE) | Comments |
|  | **Y90-TARE** | **Standard treatment^a^** |  |  |  |  |
| **Overall survival** | Estimated median in months | | HR=0.99  (0.81-1.21) | 1380 in 6 studies | Low | … |
|  | 15.5 | 15 |  |  |  |  |
| **Progression-free survival** | Estimated median in months | | HR=0.96  (0.83-1.11) | 843 in 3 studies | Very low | … |
|  | 21.5 | 20.5 |  |  |  |  |
| **Time to progression** | Estimated median in months | | HR=0.49  (0.20-1.21) | 497 in 4 studies | Very low | … |
|  | 19.5 | 9.5 |  |  |  |  |
| **Disease control rate** | Estimated proportions | | RR=0.95  (0.77-1.16) | 893 in 5 studies | Very low | … |
|  | 491/1000 | 562/1000 |  |  |  |  |
| **Grade** $\boldsymbol{\geq}$**3 adverse events** | Estimated proportions | | RR=0.64  (0.45-0.92) | 1245 in 7 studies | Low | This effect was associated with the use of sorafenib as a comparator, the absence of an active co-intervention and a balanced proportion of the different BCLC stages. |
|  | 284/1000 | 438/1000 |  |  |  |  |
| **Incidence of gastro-intestinal ulcers** | Estimated proportions | | RR=2.73  (0.70 -10.59) | 1105 in 4 studies | Very low | … |
|  | 13/1000 | 3/1000 |  |  |  |  |

^a^Estimated median survival for standard treatment for OS, progression-free survival and time to progression are assumed median survival which we calculated from weighted median survival from the trials.
