# Supplementary material for: Effect of Yttrium-90 transarterial radioembolization in patients with non-surgical hepatocellular carcinoma: A systematic review and meta-analysis
Source: PLoS One. 2021 Mar 4;16(3):e0247958. doi: 10.1371/journal.pone.0247958 (PMC7932100; doi:10.1371/journal.pone.0247958)
Supplement: S1 Appendix — (DOCX) [file pone.0247958.s004.docx]

**S1 Appendix**: Assessment of the risk of bias of included trials for all outcomes.

**Table A:** Summary table of the assessment of risk of bias for overall survival according to the RoB 2 tool.

| **Outcome assessed for risk of bias:** Overall survival | | | | | | | | | |
| --- | --- | --- | --- | --- | --- | --- | --- | --- | --- |
| **Study ID** | **Trial name** | **Experimental intervention** | **Comparator intervention** | **Domains of the Revised Cochrane risk-of-bias tool for randomized trials**  (RoB 2 tool) | | | | | **Overall RoB** |
|  |  |  |  | **Randomization process** | **Effect of assignment to intervention** | **Missing outcome data** | **Measurement of the outcome** | **Selection of the reported result** |  |
| **Chow 2018** | SIRveNIB | Y90-TARE | Sorafenib | Low | Low | Low | Low | Low | Low |
| **Dhondt 2020** | TRACE | Y90-TARE | debTACE | Low | Low | Low | Low | Low | Low |
| **Kolligs 2015** | SIRTACE | Y90-TARE | cTACE | Low | Low | Low | Low | Some concerns | Some concerns |
| **Mazzaferro 2019** | YES-P | Y90-TARE | Sorafenib | Low | High | High | Low | Low | High |
| **Pitton 2015** |  | Y90-TARE | debTACE | Low | Low | Low | Low | Some concerns | Some concerns |
| **Ricke 2019** | SORAMIC | Y90-TARE + Sorafenib | Sorafenib | Low | High | Low | Low | Low | High |
| **Salem 2016** | PREMIERE | Y90-TARE | cTACE | Low | Low | Low | Low | Low | Low |
| **Vilgrain 2017** | SARAH | Y90-TARE | Sorafenib | Low | Low | Low | Low | Low | Low |

**Table B:** Summary table of the assessment of risk of bias for progression-free survival according to the RoB 2 tool.

| **Outcome assessed for risk of bias:** Progression-free survival | | | | | | | | | |
| --- | --- | --- | --- | --- | --- | --- | --- | --- | --- |
| **Study ID** | **Trial name** | **Experimental intervention** | **Comparator intervention** | **Domains of the Revised Cochrane risk-of-bias tool for randomized trials**  (RoB 2 tool) | | | | | **Overall RoB** |
|  |  |  |  | **Randomization process** | **Effect of assignment to intervention** | **Missing outcome data** | **Measurement of the outcome** | **Selection of the reported result** |  |
| **Chow 2018** | SIRveNIB | Y90-TARE | Sorafenib | Low | Low | Low | Low | Low | Low |
| **Kolligs 2015** | SIRTACE | Y90-TARE | cTACE | Low | Low | Low | High | Some concerns | High |
| **Pitton 2015** |  | Y90-TARE | debTACE | Low | Low | Low | Some concerns | Some concerns | Some concerns |
| **Vilgrain 2017** | SARAH | Y90-TARE | Sorafenib | Low | Low | High | Some concerns | High | High |

**Table C:** Summary table of the assessment of risk of bias for time to progression according to the RoB 2 tool.

| **Outcome assessed for risk of bias:** Time to progression | | | | | | | | | |
| --- | --- | --- | --- | --- | --- | --- | --- | --- | --- |
| **Study ID** | **Trial name** | **Experimental intervention** | **Comparator intervention** | **Domains of the Revised Cochrane risk-of-bias tool for randomized trials**  (RoB 2 tool) | | | | | **Overall RoB** |
|  |  |  |  | **Randomization process** | **Effect of assignment to intervention** | **Missing outcome data** | **Measurement of the outcome** | **Selection of the reported result** |  |
| **Chow 2018** | SIRveNIB | Y90-TARE | Sorafenib | Low | Low | Low | Low | Low | Low |
| **Dhondt 2020** | TRACE | Y90-TARE | debTACE | Low | Low | Low | Some concerns | Low | Some concerns |
| **Mazzaferro 2019** | YES-P | Y90-TARE | Sorafenib | Low | High | High | High | Low | High |
| **Pitton 2015** |  | Y90-TARE | debTACE | Low | Low | Low | Some concerns | Some concerns | Some concerns |
| **Salem 2016** | PREMIERE | Y90-TARE | cTACE | Low | Low | Low | Low | Low | Low |

**Table D:** Summary table of the assessment of risk of bias for disease control rate according to the RoB 2 tool.

| **Outcome assessed for risk of bias:** Disease control rate | | | | | | | | | |
| --- | --- | --- | --- | --- | --- | --- | --- | --- | --- |
| **Study ID** | **Trial name** | **Experimental intervention** | **Comparator intervention** | **Domains of the Revised Cochrane risk-of-bias tool for randomized trials**  (RoB 2 tool) | | | | | **Overall RoB** |
|  |  |  |  | **Randomization process** | **Effect of assignment to intervention** | **Missing outcome data** | **Measurement of the outcome** | **Selection of the reported result** |  |
| **Chow 2018** | SIRveNIB | Y90-TARE | Sorafenib | Low | Low | Low | Low | Low | Low |
| **Kolligs 2015** | SIRTACE | Y90-TARE | cTACE | Low | Low | Low | High | Some concerns | High |
| **Mazzaferro 2019** | YES-P | Y90-TARE | Sorafenib | Low | High | High | High | Some concerns | High |
| **Salem 2016** | PREMIERE | Y90-TARE | cTACE | Low | Low | Some concerns | Low | Some concerns | Some concerns |
| **Vilgrain 2017** | SARAH | Y90-TARE | Sorafenib | Low | Low | High | Some concerns | High | High |

**Table E:** Summary table of the assessment of risk of bias for grade $\geq$3 adverse events rate and incidence of gastro-intestinal ulcers according to the RoB 2 tool.

| **Outcome assessed for risk of bias:** Adverse events and gastro-intestinal ulcers | | | | | | | | | |
| --- | --- | --- | --- | --- | --- | --- | --- | --- | --- |
| **Study ID** | **Trial name** | **Experimental intervention** | **Comparator intervention** | **Domains of the Revised Cochrane risk-of-bias tool for randomized trials**  (RoB 2 tool) | | | | | **Overall RoB** |
|  |  |  |  | **Randomization process** | **Effect of adhering to intervention** | **Missing outcome data** | **Measurement of the outcome** | **Selection of the reported result** |  |
| **Chow 2018** | SIRveNIB | Y90-TARE | Sorafenib | Low | Low | Low | Low | Low | Low |
| **Dhondt 2020** | TRACE | Y90-TARE | debTACE | Low | Low | Low | Low | Low | Low |
| **Kolligs 2015** | SIRTACE | Y90-TARE | cTACE | Low | Low | Low | Low | Some concerns | Some concerns |
| **Mazzaferro 2019** | YES-P | Y90-TARE | Sorafenib | Low | Low | Low | Low | Low | Low |
| **Ricke 2019** | SORAMIC | Y90-TARE + Sorafenib | Sorafenib | Low | Low | Low | Low | Low | Low |
| **Salem 2016** | PREMIERE | Y90-TARE | cTACE | Low | Low | Low | Low | Low | Low |
| **Vilgrain 2017** | SARAH | Y90-TARE | Sorafenib | Low | Low | Low | Some concerns | Low | Some concerns |
