# Supplementary material for: Effect of Yttrium-90 transarterial radioembolization in patients with non-surgical hepatocellular carcinoma: A systematic review and meta-analysis
Source: PLoS One. 2021 Mar 4;16(3):e0247958. doi: 10.1371/journal.pone.0247958 (PMC7932100; doi:10.1371/journal.pone.0247958)
Supplement: S2 Appendix — (DOCX) [file pone.0247958.s005.docx]

**S2 Appendix:** Quality of evidence according to the GRADE framework

**Table A:** Quality of evidence of the primary outcome according to GRADE

**IMPACT OF Y90-TARE TREATMENT STRATEGIES IN PATIENTS WITH NON-SURGICAL HEPATOCELLULAR CARCINOMA**

**Patient or population:** patients with non-surgical hepatocellular carcinoma

**Intervention:** Y90-TARE

**Comparison:** standard of care treatment

| **Assessment of quality of evidence according to GRADE** | | | | | | | | **Number of participants** | | **Effect**  **estimate** | **Quality of evidence** | **Importance** |
| --- | --- | --- | --- | --- | --- | --- | --- | --- | --- | --- | --- | --- |
| # studies | Study design | Risk of bias | Indirectness | Inconsistency | Imprecision | Publication bias | Other | Y90-TARE | Standard treatment | HR  (95% CI) |  |  |
| **Overall survival** | | | | | | | | | | | | |
| 6* | RCT | Not serious^a^ | Not serious | Serious^b^ | Serious^c^ | Not assessable | None | 703 | 677 | 0·99  (0·81-1·21) | Low | High |

*The SIRTACE (Kolligs 2015) and YES-P (Mazzaferro 2019) trials presented partially reported data for OS and do not contribute to the effect estimate for OS.

**Author(s):** Lemieux S, Buies A

**Comments:**

1. According to the Revised Cochrane risk-of-bias tool for randomized trials (RoB 2), we judged most studies as “low” risk of bias (four out of six), one trial had “some concerns” due to absence of protocol and another was judged as “high risk” due to effect of assignment to intervention. Of the two studies that were not meta-analyzed, one was at high risk and the other had some concerns.
2. We downgraded the certainty of evidence by one level due to inconsistency.
3. We downgraded the certainty of evidence by one level because the 95% CI (0·38–1·04) includes both “no effect” and appreciable benefit (HR <0·75).

**Table B:** Quality of evidence of progression-free survival and time to progression according to GRADE

**IMPACT OF Y90-TARE TREATMENT STRATEGIES IN PATIENTS WITH NON-SURGICAL HEPATOCELLULAR CARCINOMA**

**Patient or population:** patients with non-surgical hepatocellular carcinoma

**Intervention:** Y90-TARE

**Comparison:** standard of care treatment

| **Assessment of quality of evidence according to GRADE** | | | | | | | | **Number of participants** | | **Effect**  **estimate** | **Quality of evidence** | **Importance** |
| --- | --- | --- | --- | --- | --- | --- | --- | --- | --- | --- | --- | --- |
| # of studies | Study design | Risk of bias | Indirectness | Inconsistency | Imprecision | Publication bias | Other | Y90-TARE | Standard treatment | HR  (95% CI) |  |  |
| **Progression-free survival** | | | | | | | | | | | | |
| 3† | RCT | Very serious^a^ | Not serious | Not serious^b^ | Serious | Not assessable | None | 431 | 412 | 0·96  (0·83-1·11) | Very low | Moderate |
| **Time to progression** | | | | | | | | | | | | |
| 4* | RCT | Serious^c^ | Not serious | Serious^d^ | Serious^e^ | Not assessable | None | 250 | 247 | 0·49  (0·20-1·21) | Very low | Moderate |

† The SIRTACE trial (Kolligs 2015) presented partially reported data and is therefore not included in the effect estimate for progression-free survival.

* The YES-P trial (Mazzaferro 2019) presented partially reported data and is therefore not included in the effect estimate for time to progression.

**Author(s):** Lemieux S, Buies A

**Comments:**

1. We downgraded the certainty of evidence by two levels for within-trial risk of bias (one trial as “some concerns” and and one trial as “high” risk of bias).
2. I^2^ = 0%.
3. We downgraded the certainty of evidence by one level for within-trial risk of bias (two studies had “some concerns” due to absence of blinding of outcome assessors on radiological assessment. The only trial at “high” risk is not included in the effect, due to absence of data that can be meta-analyzed).
4. We downgraded the certainty of evidence by one level for inconsistency (I^2^ = 81%). Although, heterogeneity dropped in the subgroup of the type of Y90 microsphere (glass; 2 trials; I^2^ = 0%; versus resin; 2 trials; I^2^ = 0%), we concluded that this is imprecise due the few trials in each subgroup and possible confounding factors.
5. We downgraded the certainty of evidence by one level for imprecision (the 95% CI (0·20–1·21) includes both “no effect” and appreciable benefit (HR <0·75).

**Table C:** Quality of evidence of disease control rate according to GRADE

**IMPACT OF Y90-TARE TREATMENT STRATEGIES IN PATIENTS WITH NON-SURGICAL HEPATOCELLULAR CARCINOMA**

**Patient or population:** patients with non-surgical hepatocellular carcinoma

**Intervention:** Y90-TARE

**Comparison:** standard of care treatment

| **Assessment of quality of evidence according to GRADE** | | | | | | | | **Number of participants** | | **Effect**  **estimate** | **Quality of evidence** | **Importance** |
| --- | --- | --- | --- | --- | --- | --- | --- | --- | --- | --- | --- | --- |
| # of studies | Study design | Risk of bias | Indirectness | Inconsistency | Imprecision | Publication bias | Other | Y90-TARE | Standard treatment | RR  (95% CI) |  |  |
| **Disease control rate** | | | | | | | | | | | | |
| 5 | RCT | Very serious^a^ | Not serious | Serious^b^ | Serious^c^ | Not assessable | None | 454 | 439 | 0·95  (0·77-1·16) | Very low | Low |

**Author(s):** Lemieux S, Buies A

**Comments:**

1. We downgraded the certainty of evidence for within-trial risk of bias (three trials at “high risk”, one trial as “some concerns” and one trial as “low” risk of bias).
2. We downgraded the certainty of evidence for inconsistency (I^2^ = 46%, which may represent moderate heterogeneity). No robust explanation for heterogeneity was found in subgroup analyses. YES-P trial (Mazzaferro 2019) did not provide timing of assessment, PREMIERE trial (Salem 2016) presented disease control rate at different moments after treatment, while the other trials presented “best tumor response” at any moment.
3. We downgraded the certainty of evidence for imprecision.

**Table D:** Quality of evidence of grade$\geq$3 adverse events and gastro-intestinal ulcer rate according to GRADE

**IMPACT OF Y90-TARE TREATMENT STRATEGIES IN PATIENTS WITH NON-SURGICAL HEPATOCELLULAR CARCINOMA**

**Patient or population:** patients with non-surgical hepatocellular carcinoma

**Intervention:** Y90-TARE

**Comparison:** standard of care treatment

| **Assessment of quality of evidence according to GRADE** | | | | | | | | **Number of participants** | | **Effect estimate** | **Quality of evidence** | **Importance** |
| --- | --- | --- | --- | --- | --- | --- | --- | --- | --- | --- | --- | --- |
| # of studies | Study design | Risk of bias | Indirectness | Inconsistency | Imprecision | Publication bias | Other | Y90-TARE | Standard treatment | RR  (95% CI) |  |  |
| **Grade** $\boldsymbol{\geq3}$ **adverse events** | | | | | | | | | | | | |
| 7 | RCT | Not serious^a^ | Not serious | Serious^b^ | Serious^c^ | Not assessable | None | 599 | 646 | 0·64  (0·45-0·92) | Low | High |
| **Incidence of gastro-intestinal ulcer** | | | | | | | | | | | | |
| 4 | RCT | Serious^d^ | Not serious | Not serious^e^ | Very serious | Not assessable | None | 528 | 575 | 2·73  (0·70-10·59) | Very low | High |

**Author(s):** Lemieux S, Buies A

**Comments:**

1. According to RoB 2, we assessed five of the seven studies at “low risk” and two had “some concerns” of bias, one due to selection of the reported result and the other due to measurement of the outcome.
2. We downgraded the certainty of evidence by one level for inconsistency (I^2^ = 66%, which may represent substantial heterogeneity).
3. We downgraded the certainty of evidence by one level for imprecision.
4. We downgraded the certainty of evidence by one level for within-trial risk of bias (two had “some concerns” of bias).
5. I^2^ = 0%.
