# Supplementary material for: Effect of Yttrium-90 transarterial radioembolization in patients with non-surgical hepatocellular carcinoma: A systematic review and meta-analysis
Source: PLoS One. 2021 Mar 4;16(3):e0247958. doi: 10.1371/journal.pone.0247958 (PMC7932100; doi:10.1371/journal.pone.0247958)
Supplement: S3 Appendix — (DOCX) [file pone.0247958.s006.docx]

**S3 Appendix:** Subgroup analyses of the secondary outcomes.

**Table A:** Predefined subgroup analyses for progression-free survival.

|  | Number of trials | Population | | Random-effects model | | Heterogeneity |
| --- | --- | --- | --- | --- | --- | --- |
|  |  | Y90-TARE | Standard treatment | Pooled HR | 95% CI | I^2^ (%) |
| **Risk of bias (according to RoB 2)** | | | | | | |
| Low | 1[23] | 182 | 178 | 0·89 | 0·71-1·12 | N/A |
| Some concerns | 1[27] | 12 | 12 | 0·81 | 0·35-1·86 | N/A |
| High | 1[30] | 237 | 222 | 1·03 | 0·85-1·25 | N/A |
| **Type of comparator** | | | | | | |
| TACE | 1[27] | 12 | 12 | 0·81 | 0·35-1·86 | N/A |
| Sorafenib | 2[23,30] | 419 | 400 | 0·97 | 0·84-1·12 | 0% |
| **Disease stage** | | | | | |  |
| Mostly BCLC A-B | 2[23,27] | 194 | 190 | 0·88 | 0·71-1·10 | 0% |
| Mostly BCLC C | 1[30] | 237 | 222 | 1·03 | 0·85-1·25 | N/A |
| **Proportion of BCLC stages A and B** | | | | | | |
| <33% | 1[30] | 237 | 222 | 1·03 | 0·85-1·25 | N/A |
| 33-66% | 1[23] | 182 | 178 | 0·89 | 0·71-1·12 | N/A |
| >66% | 1[27] | 12 | 12 | 0·81 | 0·35-1·86 | N/A |
| **Proportion of BCLC stage C** | | | | | | |
| <33% | 1[27] | 12 | 12 | 0·81 | 0·35-1·86 | N/A |
| 33-66% | 1[23] | 182 | 178 | 0·89 | 0·71-1·12 | N/A |
| >66% | 1[30] | 237 | 222 | 1·03 | 0·85-1·25 | N/A |
| **Portal vein invasion or tumor thrombosis** | | | | | | |
| Majority | 1[30] | 237 | 222 | 1·03 | 0·85-1·25 | N/A |
| Minority | 1[23] | 182 | 178 | 0·89 | 0·71-1·12 | N/A |
| None | 1[27] | 12 | 12 | 0·81 | 0·35-1·86 | N/A |

Y90-TARE= Yttrium-90 transarterial radioembolization. HR=hazard ratio. CI=confidence interval.

Presence of co-treatment is not reported because all three trials did not use a co-treatment.

Type of Y-90 microsphere is not reported because all three trials used resin microspheres.

**Table B:** Predefined subgroup analyses for time to progression.

|  | Number of trials | Population | | Random-effects model | | Heterogeneity |
| --- | --- | --- | --- | --- | --- | --- |
|  |  | Y90-TARE | Standard treatment | Pooled HR | 95% CI | I^2^ (%) |
| **Risk of bias (according to RoB 2)** | | | | | | |
| Low | 2[23,29] | 206 | 199 | 0·38 | 0·06-2·58 | 84% |
| Some concerns | 2[24,27] | 44 | 48 | 0·56 | 0·12-2·67 | 79% |
| High | 0 |  |  |  |  |  |
| **Type of comparator** | | | | | | |
| TACE | 3[24,27,29] | 68 | 69 | 0·36 | 0·11-1·21 | 70% |
| Sorafenib | 1[23] | 182 | 178 | 0·88 | 0·70-1·10 | N/A |
| **Proportion of BCLC stages A and B** | | | | | | |
| <33% | 0 |  |  |  |  |  |
| 33-66% | 1[23] | 182 | 178 | 0·88 | 0·70-1·10 | N/A |
| >66% | 3[24,27,29] | 68 | 69 | 0·36 | 0·11-1·21 | 70% |
| **Proportion of BCLC stage C** | | | | | | |
| <33% | 3[24,27,29] | 68 | 69 | 0·36 | 0·11-1·21 | 70% |
| 33-66% | 1[23] | 182 | 178 | 0·88 | 0·70-1·10 | N/A |
| >66% | 0 |  |  |  |  |  |
| **Portal vein invasion or tumor thrombosis** | | | | | | |
| Majority | 0 |  |  |  |  |  |
| Minority | 1[23] | 182 | 178 | 0·88 | 0·70-1·10 | N/A |
| None | 3[24,27,29] | 68 | 69 | 0·36 | 0·11-1·21 | 70% |
| **Type of Y90 microsphere** | | | | | | |
| Resin | 2[23,27] | 194 | 190 | 0·89 | 0·71-1·11 | 0% |
| Glass | 2[24,29] | 56 | 57 | 0·23 | 0·12-0·45 | 0% |

Y90-TARE= Yttrium-90 transarterial radioembolization. HR=hazard ratio. CI=confidence interval. N/A=not applicable.

Disease stage and co-treatment subgroups are not presented because all four trials had populations of mostly BCLC stages A and B patients and did not use a co-treatment.

**Table C:** Predefined subgroup analyses for disease control rate.

|  | Number of trials | Population | | Random-effects model | | Heterogeneity |
| --- | --- | --- | --- | --- | --- | --- |
|  |  | Y90-TARE | Standard treatment | Pooled RR | 95% CI | I^2^ (%) |
| **Risk of bias (according to RoB 2)** | | | | | | |
| Low | 1[23] | 182 | 178 | 1·30 | 0·83-2·02 | N/A |
| Some concerns | 1[29] | 7 | 8 | 0·98 | 0·77-1·24 | N/A |
| High | 3[25,26,30] | 265 | 253 | 0·84 | 0·68-1·03 | 14% |
| **Type of comparator** | | | | | | |
| TACE | 2[25,29] | 20 | 23 | 1·16 | 0·85-1·58 | 0% |
| Sorafenib | 3[23,26,30] | 434 | 416 | 0·86 | 0·70-1·05 | 37% |
| **Disease stage** | | | | | | |
| Mostly BCLC A-B | 3[23,25,29] | 202 | 201 | 1·04 | 0·86-1·26 | 0% |
| Mostly BCLC C | 2[26,30] | 252 | 238 | 0·79 | 0·68-0·91 | 0% |
| **Proportion of BCLC stages A and B** | | | | | | |
| <33% | 2[26,30] | 252 | 238 | 0·79 | 0·68-0·91 | 0% |
| 33-66% | 1[23] | 182 | 178 | 0·98 | 0·77-1·25 | N/A |
| >66% | 2[25,29] | 20 | 23 | 1·16 | 0·85-1·58 | 0% |
| **Proportion of BCLC stage C** | | | | | | |
| <33% | 2[25,29] | 20 | 23 | 1·16 | 0·85-1·58 | 0% |
| 33-66% | 1[23] | 182 | 178 | 0·98 | 0·77-1·25 | N/A |
| >66% | 2[26,30] | 252 | 238 | 0·79 | 0·68-0·91 | 0% |
| **Portal vein invasion or tumor thrombosis** | | | | | | |
| Majority | 2[26,30] | 252 | 238 | 0·79 | 0·68-0·91 | 0% |
| Minority | 1[23] | 182 | 178 | 0·98 | 0·77-1·25 | N/A |
| None | 2[25,29] | 20 | 23 | 1·16 | 0·85-1·58 | 0% |
| **Type of Y90 microsphere** | | | | | | |
| Resin | 3[23,25,30] | 432 | 415 | 0·88 | 0·73-1·06 | 0% |
| Glass | 2[26,29] | 22 | 24 | 1·32 | 0·85-2·05 | 0% |

Y90-TARE= Yttrium-90 transarterial radioembolization. HR=hazard ratio. CI=confidence interval.

Presence of co-treatment is not presented because all five trials used no co-treatment.

**Table D:** Predefined subgroup analyses for grade $\geq$3 adverse events

|  | Number of trials | Population | | Random-effects model | | Heterogeneity |
| --- | --- | --- | --- | --- | --- | --- |
|  |  | Y90-TARE | Standard treatment | Pooled RR | 95% CI | I^2^ (%) |
| **Risk of bias (according to RoB 2)** | | | | | | |
| Low | 5[23,24,26,28,29] | 360 | 415 | 0·57 | 0·32-1·04 | 76% |
| Some concerns | 2[25,30] | 239 | 231 | 0·75 | 0·37-1·50 | 28% |
| High | 0 |  |  |  |  |  |
| **Type of comparator** | | | | | | |
| TACE | 3[24,25,29] | 69 | 70 | 0·87 | 0·56-1·34 | 0% |
| Sorafenib | 4[23,26,28,30] | 530 | 576 | 0·53 | 0·32-0·88 | 81% |
| **Co-treatment** | | | | | | |
| Systemic | 1[28] | 159 | 182 | 0·92 | 0·64-1·31 | N/A |
| None | 6[19-22,25,26] | 440 | 464 | 0·58 | 0·37-0·91 | 65% |
| **Disease stage** | | | | | | |
| Mostly BCLC A-B | 4[23-25,29] | 199 | 232 | 0·65 | 0·31-1·36 | 72% |
| Mostly BCLC C | 3[26,28,30] | 400 | 414 | 0·69 | 0·45-1·07 | 67% |
| **Proportion of BCLC stages A and B** | | | | | | |
| <33% | 3[26,28,30] | 400 | 414 | 0·69 | 0·45-1·07 | 67% |
| 33-66% | 1[23] | 130 | 162 | 0·30 | 0·18-0·50 | N/A |
| >66% | 2[25,29] | 37 | 34 | 1·01 | 0·41-2·48 | 0% |
| **Proportion of BCLC stage C** | | | | | | |
| <33% | 3[24,25,29] | 69 | 70 | 0·87 | 0·56-1·34 | 0% |
| 33-66% | 1[23] | 130 | 162 | 0·30 | 0·18-0·50 | N/A |
| >66% | 3[26,28,30] | 400 | 414 | 0·69 | 0·45-1·07 | 67% |
| **Portal vein invasion or tumor thrombosis** | | | | | | |
| Majority | 2[26,30] | 241 | 232 | 0·36 | 0·07-1·82 | 67% |
| Minority | 2[23,28] | 289 | 344 | 0·53 | 0·18-1·61 | 92% |
| None | 4[23,25,29] | 69 | 70 | 0·87 | 0·56-1·34 | 0% |
| **Type of Y90 microsphere** | | | | | | |
| Resin | 4[23,25,28,30] | 528 | 575 | 0·63 | 0·40-1·01 | 78% |
| Glass | 3[24,26,29] | 71 | 71 | 0·61 | 0·25-1·45 | 52% |

Y90-TARE= Yttrium-90 transarterial radioembolization. HR=hazard ratio. CI=confidence interval.

**Table E:** Predefined subgroup analyses for rates of gastro-intestinal ulcers.

|  | Number of trials | Population | | Random-effects model | | Heterogeneity |
| --- | --- | --- | --- | --- | --- | --- |
|  |  | Y90-TARE | Standard treatment | Pooled HR | 95% CI | I^2^ (%) |
| **Risk of bias (according to RoB 2)** | | | | | | |
| Low | 2[23,30] | 289 | 344 | 4·67 | 0·52-42·05 | 0% |
| Some concerns | 2[25,28] | 240 | 232 | 1·96 | 0·35-10·98 | 0% |
| High |  |  |  |  |  |  |
| **Type of comparator** | | | | | | |
| TACE | 1[25] | 14 | 16 | 1·14 | 0·08-16·63 | N/A |
| Sorafenib | 3[23,28,30] | 515 | 560 | 3·68 | 0·76-17·77 | 0% |
| **Co-treatment** | | | | | | |
| Systemic | 1[28] | 159 | 182 | 5·72 | 0·28-118·24 | N/A |
| None | 3[23,25,30] | 370 | 394 | 2·26 | 0·50-10·33 | 0% |
| **Disease stage** | | | | | | |
| Mostly BCLC A-B | 2[23,25] | 144 | 178 | 1·86 | 0·24-14·96 | 0% |
| Mostly BCLC C | 2[28,30] | 385 | 398 | 3·67 | 0·60-22·39 | 0% |
| **Proportion of BCLC stages A and B** | | | | | | |
| <33% | 2[28,30] | 385 | 398 | 3·67 | 0·60-22·39 | 0% |
| 33-66% | 2[23,25] | 144 | 178 | 1·86 | 0·24-14·96 | 0% |
| >66% |  |  |  |  |  |  |
| **Proportion of BCLC stage C** | | | | | | |
| <33% | 1[25] | 14 | 16 | 1·14 | 0·08-16·63 | N/A |
| 33-66% | 1[23] | 130 | 162 | 3·73 | 0·15-90·88 | N/A |
| >66% | 2[28,30] | 385 | 398 | 3·67 | 0·60-22·39 | 0% |
| **Portal vein invasion or tumor thrombosis** | | | | | | |
| Majority | 1[30] | 226 | 216 | 2·87 | 0·30-27·35 | N/A |
| Minority | 2[23,28] | 289 | 344 | 4·67 | 0·52-42·05 | 0% |
| None | 1[25] | 14 | 16 | 1·14 | 0·08-16·63 | N/A |

Y90-TARE= Yttrium-90 transarterial radioembolization. HR=hazard ratio. CI=confidence interval. N/E=not estimable. N/A=not applicable.

Type of Y90 microsphere is not presented because all four trials used resin microspheres.

For Kolligs 2015 (SIRTACE trial)[25], we used Y+1/n+1 to express the risk ratio, due to the value “Y=0” in both groups.
