# Supplementary material for: Effect of Yttrium-90 transarterial radioembolization in patients with non-surgical hepatocellular carcinoma: A systematic review and meta-analysis
Source: PLoS One. 2021 Mar 4;16(3):e0247958. doi: 10.1371/journal.pone.0247958 (PMC7932100; doi:10.1371/journal.pone.0247958)
Supplement: S1 Text — (DOCX) [file pone.0247958.s007.docx]

**S1 Text:** Full search strategy.

**PubMed (14-04-2020)**

**No database limit**

| **Concepts** | **#** | **Search strategy** | **Results** |
| --- | --- | --- | --- |
| Hepatocellular carcinoma | 1 | carcinoma, hepatocellular[Mesh Terms] |  |
|  | 2 | Hepatocellular Carcinoma*[TIAB] |  |
|  | 3 | Liver Cell Carcinoma*[TIAB] |  |
|  | 4 | Liver Cancer*[TIAB] |  |
|  | 5 | Hepatoma*[TIAB] |  |
|  | 6 | Hepatic Carcinoma*[TIAB] |  |
|  | 7 | Hepatic Cell Carcinoma*[TIAB] |  |
|  | 8 | Hepatocarcinoma*[TIAB] |  |
|  | 9 | Liver Carcinoma*[TIAB] |  |
|  | 10 | Malignant Hepatoma*[TIAB] |  |
| Hepatocellular carcinoma combined | 11 | #1 OR #2 OR #3 OR #4 OR #5 OR #6 OR #6 OR #8 OR #9 OR #10 | 144 187 |
| Y90-TARE | 12 | Yttrium-90[Supplementary Concept] |  |
|  | 13 | Yttrium-90 Macroaggregated Albumin[Supplementary Concept] |  |
|  | 14 | Yttrium Radioisotopes[MeSH] |  |
|  | 15 | Embolization, Therapeutic[MeSH] |  |
|  | 16 | Y-90[TIAB] |  |
|  | 17 | 90Y[TIAB] |  |
|  | 18 | 90 Y [TIAB] |  |
|  | 19 | Y90[TIAB] |  |
|  | 20 | 90 Yttrium[TIAB] |  |
|  | 21 | radioactive yttrium[tiab] |  |
|  | 22 | Radioyttrium[TIAB] |  |
|  | 23 | Yttrium 90[TIAB] |  |
|  | 24 | Selective Internal Radiotherapy*[TIAB] |  |
|  | 25 | Selective Internal Radiation therap*[TIAB] |  |
|  | 26 | Arterial Radioemboli*[TIAB] |  |
|  | 27 | Transarterial radioemboli*[TIAB] |  |
| Y90-TARE combined | 28 | #12 OR #13 OR #14 OR #15 OR #16 OR #17 OR #18 OR #19 OR #20 OR #21 OR #22 OR #23 OR #24 OR #25 OR #26 OR #27 | 46 096 |
| Randomized clinical trials | 29 | Randomized Controlled Trial[pt] |  |
|  | 30 | Controlled Clinical Trial[pt] |  |
|  | 31 | Randomized[TIAB] |  |
|  | 32 | Placebo[TIAB] |  |
|  | 33 | Clinical Trials as topic[mesh:noexp] |  |
|  | 34 | Randomly[TIAB] |  |
|  | 35 | Trial [ti] |  |
| Randomized clinical trials combined | 36 | #29 OR #30 OR #31 OR #32 OR #33 OR #34 OR #35 | 1 288 220 |
| Combination of concepts | 37 | #11 AND #28 AND #36 | 734 |
| Human study only | 38 | #37 NOT (animals[mh] NOT humans[mh]) | 708 |

**Embase.com (14-04-2020)**

**Limits:** Embase database only

| **Concepts** | **#** | **Search strategy** | **Results** |
| --- | --- | --- | --- |
| Hepatocellular carcinoma | 1 | ‘liver cell carcinoma’/exp |  |
|  | 2 | ‘hepatocellular carcinoma*’:ti,ab,kw |  |
|  | 3 | ‘liver cell carcinoma*’:ti,ab,kw |  |
|  | 4 | ‘liver cancer*’:ti,ab,kw |  |
|  | 5 | hepatoma*:ti,ab,kw |  |
|  | 6 | ‘hepatic carcinoma*’:ti,ab,kw |  |
|  | 7 | "hepatocarcinoma*":ti,ab,kw |  |
|  | 8 | "hepatic cell carcinoma*":ti,ab,kw |  |
|  | 9 | ‘liver carcinoma*’:ti,ab,kw |  |
|  | 10 | ‘malignant hepatoma*’:ti,ab,kw |  |
| Hepatocellular carcinoma combined | 11 | #1 OR #2 OR #3 OR #4 OR #5 OR #6 OR #7 OR #8 OR #9 OR #10 | 8 068 |
| Y90-TARE | 12 | ‘yttrium 90’/exp |  |
|  | 13 | ‘yttrium’/exp |  |
|  | 14 | ‘radioembolization’/exp |  |
|  | 15 | ‘radioembolization induced liver disease’/exp |  |
|  | 16 | ‘y 90’:ti,ab,kw |  |
|  | 17 | 90y:ti,ab,kw |  |
|  | 18 | Y90:ti,ab,kw |  |
|  | 19 | ’90 yttrium’:ti,ab,kw |  |
|  | 20 | ‘radioactive yttrium’:ti,ab,kw |  |
|  | 21 | Radioyttrium:ti,ab,kw |  |
|  | 22 | ‘yttrium 90’:ti,ab,kw |  |
|  | 23 | ‘selective internal radiotherapy*’:ti,ab,kw |  |
|  | 24 | ‘selective internal radiation therap*’:ti,ab,kw |  |
|  | 25 | ‘arterial radioemboli*’:ti,ab,kw |  |
|  | 26 | ‘transarterial radioemboli*’:ti,ab,kw |  |
| Y-90 combined | 27 | #12 OR #13 OR #14 OR #15 OR #16 OR #17 OR #18 OR #19 OR #20 OR #21 OR #22 OR #23 OR #24 OR #25 OR #26 | 16 702 |
| Randomized clinical trial | 28 | ‘crossover procedure’:de |  |
|  | 29 | ‘double-blind procedure’:de |  |
|  | 30 | ‘randomized controlled trial’:de |  |
|  | 31 | ‘single-blind procedure’:de |  |
|  | 32 | random*:de,ab,ti |  |
|  | 33 | factorial*:de,ab,ti |  |
|  | 34 | crossover*:de,ab,ti |  |
|  | 35 | (cross NEXT/1 over*):de,ab,ti |  |
|  | 36 | Placebo*:de,ab,ti |  |
|  | 37 | (doubl* NEAR/1 blind*):de,ab,ti |  |
|  | 38 | (singl* NEAR/1 blind*):de,ab,ti |  |
|  | 39 | Assign*:de,ab,ti |  |
|  | 40 | Allocate*:de,ab,ti |  |
|  | 41 | Volunteer*:de,ab,ti |  |
| Randomized clinical trial combined | 42 | #28 OR #29 OR #30 OR #31 OR #32 OR #33 OR #34 OR #35 OR #36 OR #37 OR #38 OR #39 OR #40 OR #41 | 2 483 978 |
| Combination of concepts | 43 | #11 AND #27 AND #42 | 400 |
| Human study only | 44 | #43 NOT ('animal'/exp NOT “human”/exp) | 397 |
| Embase database only | 45 | #44 AND [embase]/lim NOT ([embase]/lim AND [medline]/lim) | 178 |

**Cochrane (14-04-2020)**

**Limit:** trials only

| **Concepts** | **#** | **Search strategy** | **Results** |
| --- | --- | --- | --- |
| Hepatocellular carcinoma | 1 | "Hepatocellular Carcinoma*":ti,ab |  |
|  | 2 | "liver cell carcinoma*":ti,ab |  |
|  | 3 | “liver cancer*”:ti,ab |  |
|  | 4 | hepatoma*:ti,ab |  |
|  | 5 | "hepatic carcinoma*":ti,ab |  |
|  | 6 | "hepatic cell carcinoma":ti,ab |  |
|  | 7 | hepatocarcinoma*:ti,ab |  |
|  | 8 | "liver carcinoma*":ti,ab |  |
|  | 9 | "malignant hepatoma*":ti,ab |  |
|  | 10 | [mh "carcinoma, hepatocellular"] |  |
| Hepatocellular combined | 11 | {OR #1-#10} | 5271 |
| Y90-TARE | 12 | "Y-90":ti,ab |  |
|  | 13 | 90Y:ti,ab |  |
|  | 14 | "90 Y":ti,ab |  |
|  | 15 | Y90:ti,ab |  |
|  | 16 | "90 yttrium":ti,ab |  |
|  | 17 | "radioactive yttrium":ti,ab |  |
|  | 18 | "radioyttrium":ti,ab |  |
|  | 19 | "yttrium 90":ti,ab |  |
|  | 20 | "selective internal radiotherapy":ti,ab |  |
|  | 21 | "selective internal radiation therap*":ti,ab |  |
|  | 22 | "arterial radioemboli*":ti,ab |  |
|  | 23 | "transarterial radioemboli*":ti,ab |  |
|  | 24 | [mh "Embolization, Therapeutic"] |  |
|  | 25 | [mh "Yttrium Radioisotopes"] |  |
| Y90-TARE combined | 26 | {OR #12-#25} | 1427 |
| Combination of concepts | 27 | #11 AND #26 | 369 |
| Trials Only | 28 |  | 360 |

**Web of Science (14-06-2020)**

**Limits:** Science Citation Index Expanded (SCI-EXPANDED) --1900-present

Conference Proceedings Citation Index- Science (CPCI-S) --1990-present

Emerging Sources Citation Index (ESCI) --2005-present

| **Concepts** | **#** | **Search strategy** | **Results** |
| --- | --- | --- | --- |
| Hepatocellular carcinoma | 1 | TS=("hepatocellular carcinoma*") |  |
|  | 2 | TS=("liver cell carcinoma*") |  |
|  | 3 | TS=("liver cancer*") |  |
|  | 4 | TS=(hepatoma*) |  |
|  | 5 | TS=("hepatic carcinoma*") |  |
|  | 6 | TS=("hepatic cell carcinoma") |  |
|  | 7 | TS=( hepatocarcinoma*) |  |
|  | 8 | TS=("liver carcinoma*") |  |
| Hepatocellular carcinoma combined | 9 | #8 OR #7 OR #6 OR #5 OR #4 OR #3 OR #2 OR #1 | 177 340 |
| Y90-TARE | 10 | TS=(Y-90) |  |
|  | 11 | TS=(90Y) |  |
|  | 12 | TS=(“90 Y”) |  |
|  | 13 | TS=(Y90) |  |
|  | 14 | TS=(“90 yttrium”) |  |
|  | 15 | TS=(“radioactive yttrium”) |  |
|  | 16 | TS=(“radioyttrium”) |  |
|  | 17 | TS=(“yttrium 90”) |  |
|  | 18 | TS=(“selective internal radiotherapy”) |  |
|  | 19 | TS=(“selective internal radiation therap*”) |  |
|  | 20 | TS=(“arterial radioemboli*”) |  |
|  | 21 | TS=(“transarterial radioemboli*”) |  |
| Y90-TARE combined | 22 | #21 OR #20 OR #19 OR #18 OR #17 OR #16 OR #15 OR #14 OR #13 OR #12 OR #11 OR #10 | 9 151 |
| Randomized clinical trial | 23 | TS=(”clinical trial*”) |  |
|  | 24 | TS=(”research design”) |  |
|  | 25 | TS=(”comparative stud*”) |  |
|  | 26 | TS=(”evaluation stud*”) |  |
|  | 27 | TS=(”controlled trial*”) |  |
|  | 28 | TS=(”follow-up stud*”) |  |
|  | 29 | TS=(”prospective stud*”) |  |
|  | 30 | TS=(random*) |  |
|  | 31 | TS=(placebo*) |  |
|  | 32 | TS=(“single blind*”) |  |
|  | 33 | TS=(“double blind*”) |  |
| Randomized clinical trial combined | 34 | #33 OR #32 OR #31 OR #30 OR #29 OR #28 OR #27 OR #26 OR #25 OR #24 OR #23 | 2 576 202 |
| Combination of concepts | 35 | #34 AND #22 AND #9 | 358 |
