# Supplementary material for: Effect of Yttrium-90 transarterial radioembolization in patients with non-surgical hepatocellular carcinoma: A systematic review and meta-analysis
Source: PLoS One. 2021 Mar 4;16(3):e0247958. doi: 10.1371/journal.pone.0247958 (PMC7932100; doi:10.1371/journal.pone.0247958)
Supplement: Attachment — Submitted filename: Response To Reviewers.docx [file pone.0247958.s008.docx]

Quebec, January 21^th^, 2020

**Dr Joerg Heber,** Editor-in-Chief

**Dr Yi-Hsiang Huang**, Academic Editor

*PLOS ONE*

Dear Dr Heber and Dr Huang,

Encouraged by the reviewers’ and editors’ comments received on December 12^th^, 2020, we are submitting a revision of our manuscript titled “**Effect of Yttrium-90 transarterial radioembolization in patients with non-surgical hepatocellular carcinoma: a systematic review and meta-analysis**”. We believe we have addressed every concern and suggestion raised by the reviewers. We are thankful for the constructive feedback that we have received and believe that this revision strengthens our manuscript further and will increase the confidence of your readers in the presented results. We hope that this work will now be acceptable for publication in *PLOS ONE*.

Thank you for your consideration.

Sincerely,

Simon Lemieux, M.D., M.Sc. candidate

PGY-4 Radiology Resident

Department of Radiology and Nuclear Medicine

Université Laval

1050, Avenue de la Médecine

Québec City, Québec, Canada

Tél : 418-558-7790

[simon.lemieux.9@ulaval.ca](mailto:simon.lemieux.9@ulaval.ca)

**REVIEWERS’ COMMENTS:**

We thank the reviewers and the editors for their constructive comments, which were all addressed.

**Comments from the editors:**

1. Please ensure that your manuscript meets PLOS ONE's style requirements, including those for file naming.

**We have revised our manuscript according to PLOS ONE’s style requirements. We have also reviewed all file naming.**

2. Please amend your list of authors on the manuscript to ensure that each author is linked to an affiliation. Authors’ affiliations should reflect the institution where the work was done (if authors moved subsequently, you can also list the new affiliation stating “current affiliation:….” as necessary).

**The list of authors has been revised and modifications have been made to ensure that each author is linked to an affiliation.**

**Response to reviewer #1 comments:**

Strengths:
A. This study was conducted under an exhaustive search and unpublished data from the reference authors were requested.
B. All the enrolled studies were published RCTs and is different from prior meta-analysis studies which included cohort studies.

**Thank you for noting this.**

Weakness:
The enrolled studies were categorized into Y90-TARE vs. cTACE and Y90-TARE vs. Sorafenib initially, but in data analysis, all the studies were mixed and were divided as Y90-TARE vs. standard of care. Most subgroup analyses were also not very robust considering the limited number of trials

**We thank the reviewer for his comment. The main purpose of a meta-analysis is to synthesize treatment effects considering all available data. By pooling data from different trials, the power to detect a differential effect is increased. Trials included in a systematic review frequently present some degree of clinical heterogeneity. This recommended approach improves the ability to answer important research questions and understand the modifiers of important treatment effects, such as type of comparator treatment. We however planned subgroup analyses *a priori* to evaluate whether the type of comparator could explain the findings.**

**Response to reviewer #2 comments:**

Major comments
1. Although the methods and statistics are correct in this study, major limitation still could not be ignored in the enrolled studies because 2 of 8 trials with a high risk of bias and 2 trials with concerns of bias in analysis of overall survival. In addition, most trials were at high risk of bias or of concerns in analysis of secondary outcomes, including progression-free survival, time to progression, disease control rate, etc. Such limitations would have great impact on the results in this study. The authors would at least eliminate two trials of high risk of bias in analysis of primary outcome.

**We thank the reviewer for the thorough assessment of our work. As highlighted in our risk of bias assessment section, some of the included trials were at high risk of bias. However, rather than excluding trials, we explored the potential effect of the risk of bias in our subgroup analyses, as suggested by the Cochrane Methodology of systematic reviews and meta-analysis.[1] Subgroup analyses are in such situation more informative than sensitivity analyses where removing analyses would only inform of the remaining subgroup.**

2. Subgroup analysis according to the presence of co-treatment with systemic therapy was conducted for the rates of gastrointestinal ulcers. The authors still have better to perform similar subgroup analysis in other outcomes.

**We performed subgroup analyses for each of our outcomes when possible to do so. Some could however not be conducted due to the absence of trials in each subgroup category. Progression-free survival, time to progression, and disease control rate were outcomes which only included Y90-TARE without co-treatment. We added specifications regarding the subgroup analyses that could not be conducted in S3 Appendix (under Tables A, B, and C).**

Minor comments
1. There are many clinical evidences real-world data to support the new treatments of advanced or unresectable HCC. Therefore, the authors have better to revise the introduction “although new treatments are available, the survival benefit is still not optimal and new alternatives are sought”.

**Thank you for your comment. Although many new treatments showing promising results are available, the quality of the evidence is still not optimal to be very assertive on the matter. We therefore prefer to keep the same phrasing.**

2. Citation should be added on each trial in the table 1.

**The change has been made to Table 1.**

3. Y90-TARE seems to yield a significantly longer time to progression in the glass microsphere subgroup. The authors have to discuss this finding well.

**Thank you for your comment. We modified the first paragraph of the discussion to highlight this finding and we also put this result in context with prior knowledge in the second paragraph of the discussion.**

**REFERENCES:**

1. Boutron I, Page MJ, Higgins JPT, Altman DG, Lundh A, Hróbjartsson A. Chapter 7: Considering bias and conflicts of interest among the included studies. In: Higgins JPT, Thomas J, Chandler J, Cumpston M, Li T, Page MJ, Welch VA (editors). *Cochrane Handbook for Systematic Reviews of Interventions* version 6.1 (updated September 2020). Cochrane, 2020. Available from [www.training.cochrane.org/handbook](http://www.training.cochrane.org/handbook).
